# Multi-Scale Modeling of Doped Magnesium Hydride Nanomaterials for Hydrogen Storage Applications

**DOI:** 10.3390/nano15191470

**Published:** 2025-09-25

**Authors:** Younes Chrafih, Rubayyi T. Alqahtani, Abdelhamid Ajbar, Bilal Lamrani

**Affiliations:** 1 Lphe-Modeling and Simulation Faculty of Science, Mohammed V University in Rabat, Rabat 1014, Morocco; y.chrafih@um5r.ac.ma; 2 Department of Mathematics and Statistics, College of Science, Imam Mohammad Ibn Saud Islamic University (IMSIU), Riyadh 11432, Saudi Arabia; rtalqahtani@imamu.edu.sa; 3 Department of Chemical Engineering, College of Engineering, King Saud University, Riyadh 12372, Saudi Arabia; aajbar@ksu.edu.sa; 4 MANAPSE Lab, Faculty of Sciences, Mohammed V University in Rabat, Rabat 1014, Morocco

**Keywords:** nanostructured materials, hydrogen storage, multi-scale modeling, density functional theory (DFT), thermal management, MgH_2_ doping

## Abstract

This work presents the development of a novel multi-scale modeling framework for investigating the beneficial impact of Ti-, Zr-, and V-doped magnesium hydride nanomaterials on hydrogen storage performance. The proposed model integrates atomistic-scale simulations based on density functional theory (DFT) with system-level dynamic heat and mass transfer modeling. At the nanoscale, DFT analysis provides key thermodynamic and kinetic parameters, including reaction enthalpy, entropy, and activation energy, which are incorporated into the macroscopic model to predict the hydrogenation behavior of MgH_2_ nanostructures under realistic thermal boundary conditions. Model validation is performed through comparison with experimental data from the literature, showing excellent agreement. The DFT analysis reveals that doping MgH_2_ nanomaterials with Ti, V, and Zr modifies their thermodynamic properties, including enthalpy of formation and desorption temperature. At the reactor scale, these modifications lead to enhanced hydrogenation kinetics and improved thermal management. Compared to pristine MgH_2_, hydrogenation time is reduced by 21%, 40%, and 42% for Ti-, Zr-, and V-doped nanomaterials, respectively, while thermal energy consumption during hydrogenation decreases by ~17% for V doping. These results highlight the strong correlation between nanoscale modifications and macroscopic system performance. The proposed multi-scale model provides a powerful tool for guiding the design and optimization of advanced nanostructured hydrogen storage materials for sustainable energy applications.

## 1. Introduction

The global shift toward sustainable energy systems has intensified research efforts on hydrogen as a clean and effective medium for energy transport. Among the various hydrogen storage technologies, solid-state storage using metal hydrides is widely recognized for its high volumetric energy density, safety, and reversibility [1,2]. Magnesium hydride (MgH_2_), in particular, has received extensive attention because of to its relatively high theoretical hydrogen capacity (~7.6 wt%), low cost and abundance [3,4]. Nonetheless, its practical use is constrained by sluggish hydrogen sorption kinetics and elevated desorption temperatures, typically exceeding 300 °C [5].

**Figure 1:**
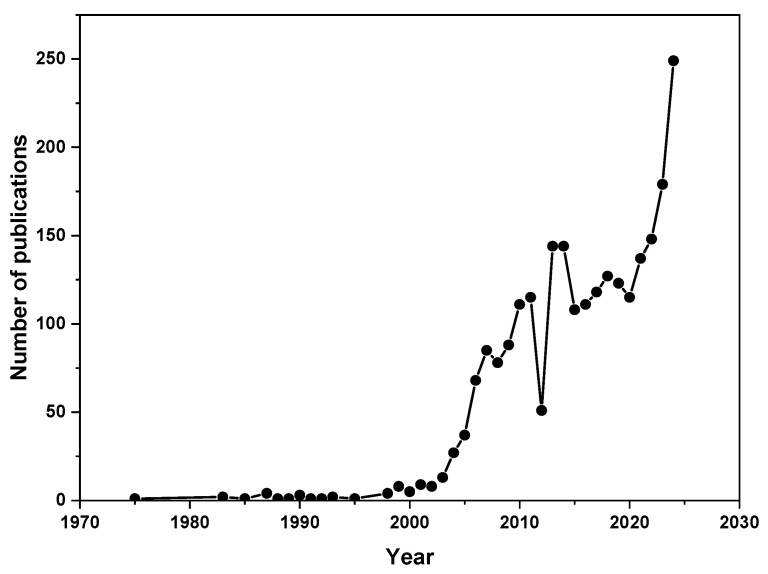
Progress of the published works on MgH_2_ for hydrogen storage (Scopus database, 10 July 2025).

This scientific interest is reflected in the steadily increasing number of publications on MgH_2_-based hydrogen storage systems over the past two decades (Figure 1). According to data retrieved from the Scopus database using the search terms “magnesium hydride” and “hydrogen storage”, research output has grown significantly, indicating the ongoing relevance of MgH_2_ in hydrogen energy research and the persistent efforts to improve its performance.

Recent advancements in hydrogen storage have shown that transition metal doping of MgH_2_ greatly improves its performance. For instance, Xiao et al. [6] demonstrated that using ball milling to add Ce_0.6_ Zr_0.4_O_2_ nanocrystals to MgH_2_ significantly reduced the beginning dehydrogenation temperature by 85 °C to 201 °C. This composite exhibited rapid hydrogen release (6.15 wt% at 270 °C) and absorption (6.3 wt% in 2 min at 50 bar and 150 °C), maintaining remarkable cycling stability exhibiting 98.9% capacity retention over the course of 20 cycles.

Additionally, MgH_2_-30 wt% TiFe_0.92_Mn_0.04_Co_0.04_ was synthesized by ball milling by Li et al. [7], demonstrating remarkable hydrogen storage capabilities. In contrast to conventional TiFe, the addition of Mn and Co lowered the activation barriers of TiFe_0.92_Mn_0.04_Co_0.04_ and increased its catalytic activity by facilitating the in situ synthesis of α-Fe.

Furthermore, Han et al. [8] examined the impact of Ti, Zr, V, and Nb doping on MgH_2_ using first-principles methods. According to these investigations, these dopants speed up the kinetics of hydrogen desorption by facilitating the transport of hydrogen atoms far from the dopant sites. Together, our results demonstrate the significant promise of Ti, Zr, V, and Nb doping to enhance MgH_2_’s usefulness as a practical hydrogen storage substance.

Magnesium hydride (MgH_2_) is an attractive material for hydrogen storage because of its high gravimetric and volumetric hydrogen capabilities (7.6 wt% and 110 g/L, respectively). It outperforms several other options, including carbon nanostructures like nanotubes and graphene, which typically have low gravimetric capacities (<1 wt%) at ambient temperature, and complicated hydrides (e.g., NaAlH_4_) with complex reaction paths and poor reversibility. While Metal–Organic Frameworks (MOFs) have large surface areas for hydrogen adsorption, their practical application is typically limited to cryogenic temperatures due to poor interactions with hydrogen molecules. MgH_2_ is an excellent choice because it is lightweight, inexpensive, and abundant. However, its primary constraints are slow kinetics and great thermodynamic stability, which necessitate high temperatures for hydrogen release and absorption [9,10,11]. The MgH_2_ lattice’s hydrogen desorption behavior can be significantly modified via the addition of vacancies to it. According to Gaztanaga et al. [12], MgH_2_ regularly shows negatively charged divacancies in addition to positively and negatively charged hydrogen vacancies. The material’s ability to store hydrogen can be substantially improved by fixing these flaws. Additionally, the material’s band gap is significantly reduced when negative and neutral hydrogen vacancies are present, improving its semiconductor qualities. German et al. [13] further demonstrated that Zr doping is more effective than Nb in reducing dehydrogenation energy. Additionally, the creation of vacancies within the Mg lattice leads to a significant decrease in energy of dehydrogenation, with vacancies in the Mg sublattice representing the arrangement that yields the lowest desorption energies. In comparison to the pure hydride, C.R. Luna et al. [14] found that adding trace amounts of Zr or Nb impurities (about 0.01 weight percent) to immaculate MgH_2_ had no discernible impact on the electron momentum density. Conversely, a significant change in electron density was observed at the vacancy site in MgH_2_ where a Mg vacancy exists. Moreover, the electron momentum density at the vacancy site is significantly altered when a substitutional impurity is present next to a Mg vacancy at the same time.

Finally, Reddad et al. [15] employed Density Functional Theory (DFT) to investigate the effects of Cu and Zn doping on the kinetic and thermodynamic properties of MgH_2_. They methodically investigate how different Cu and Zn dopant concentrations and configurations affect MgH_2_’s hydrogen desorption properties.

While numerical results have demonstrated improvements in hydrogen storage, a complete mechanistic understanding linking atomic-scale modifications to system-level behavior has remained incomplete. To bridge this gap, multi-scale modeling approaches that couple atomistic simulations with thermal and kinetic system-level models are essential. Previous literature has shown that DFT provides accurate predictions of material properties (e.g., reaction enthalpy and activation energy), while dynamic thermal modeling effectively captures the effects of heat and mass transfer in real operating conditions. In this study, we propose a novel coupled atomistic and dynamic thermal modeling framework to investigate the impact of Ti, Zr, and V doping on MgH_2_. This approach integrates DFT-calculated parameters directly into a system-level dynamic model, enabling the quantification of hydrogenation time, energy consumption, and thermal behavior. The system-level model is fundamentally based on energy and mass balances, meticulously coupled with the hydrogenation/dehydrogenation kinetic equations. It is interesting to note that in the present work, a lumped parameter approach was adopted to model the dynamic behavior of the MgH_2_-based hydrogen storage reactor. While spatially resolved finite-element or CFD models can capture local gradients in temperature, pressure, and concentration with high fidelity, they are computationally expensive and often impractical for extensive parametric studies or integration into system-level analyses. The lumped model used here provides a balance between accuracy and computational efficiency, enabling fast simulations while still reproducing the dominant thermal and kinetic trends observed in experimental studies. Such an approach is particularly valuable for early-stage design, performance optimization, and coupling with real-time control strategies, whereas distributed models remain more suitable for detailed reactor-scale optimization and scale-up considerations. The results from this integrated framework are expected to highlight the direct correlation between atomic-scale doping effects and macroscopic system performance, offering a robust tool for the design and optimization of advanced hydrogen storage systems.

The novelty of the proposed work can be summarized as:

- Novel coupled atomistic and dynamic thermal modeling framework: This study pioneers the direct, integrated application of atomistic (DFT) and system-level (dynamic thermal) models specifically for the analysis and optimization of hydrogen storage materials. Unlike previous studies that might use these methods in isolation or in a less integrated fashion, our framework establishes a seamless connection to bridge the disparate length and timescales.
- Direct correlation of atomic-scale doping with macroscopic system performance: A central innovative aspect is the explicit linkage established between the fundamental effects of specific dopants (Ti, Zr, V) on MgH_2_ at the atomic scale (e.g., changes in reaction barriers and enthalpies from DFT) and their direct impact on observable, macroscopic system-level outcomes (e.g., overall hydrogenation time, energy consumption, and thermal management). This provides a mechanistic understanding that was previously lacking.
- Comprehensive quantification of system behavior under realistic conditions: Beyond just material properties, our framework allows for the comprehensive quantification of crucial system performance indicators such as hydrogenation/dehydrogenation time, total energy consumption, and detailed thermal behavior under dynamic operating conditions. This moves beyond theoretical predictions to practical system-level performance assessment.

## 2. Multi-Scale Modeling Method

The proposed modeling approach follows a multi-scale framework, combining atomic-scale DFT calculations with macroscopic system-level dynamic modeling to analyze the hydrogen storage performance of MgH_2_ doped with Ti, V, and Zr. This methodology enables a direct link between fundamental material properties and large-scale system behavior, providing a comprehensive understanding of doping effects from the atomic level to practical applications.

At the microscopic scale, DFT calculations are employed to determine key material properties such as formation energy, hydrogen desorption enthalpy, diffusion barriers, and electronic structure. These parameters provide insight into how Ti, V, and Zr influence the thermodynamics and kinetics of hydrogen absorption and desorption in MgH_2_. The results obtained from the DFT simulations are then used as input parameters IN the macroscopic system-level model, which describes the dynamic behavior of a hydrogen storage tank using a lumped modeling approach. This model accounts for mass and heat transfer, reaction kinetics, and thermal management by using cooling/heating fluid, hydrogen uptake/release rates, and the overall performance of the storage system under realistic operating conditions.

**Figure 2:**
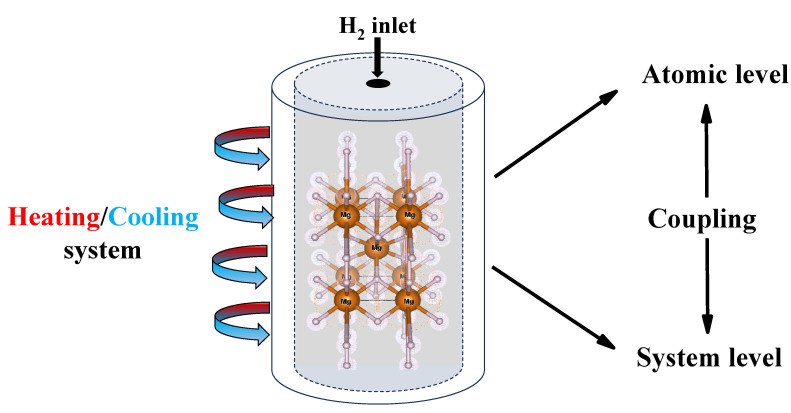
Representation of the coupling between the atomic level and the system level of MgH_2_.

By coupling the atomic-scale and system-level models, this multi-scale approach enables a direct correlation between material modifications at the microscopic level and their impact on storage performance at the macroscopic scale. This integration is crucial for optimizing MgH_2_-based hydrogen storage systems, ensuring both improved material properties and enhanced practical performance. A schematic representation of this coupling is shown in Figure 2, where the atomic-scale structure of MgH_2_ and the system-level storage configuration are illustrated.

### 2.1. Microscopic Scale: DFT Modeling of Doped MgH_2_

#### 2.1.1. Computational Theory

The Cambridge Serial Total Energy Package (CASTEP) and density functional theory serve as the foundation for all of the computations in this work [16,17]. The option guarantees energy convergence to less than 1 meV per atom. The electron wave function was expanded in plane waves up to a cutoff energy of 350 eV. The exchange-correlation functional was created based on the generalized gradient approximation (GGA) as outlined by Perdew–Burke–Ernzerhof revised for solids (PBEsol) [17,18]. Prior research has demonstrated that PBEsol is superior to PBE for examining point defects [19] and can forecast lattice constants with more accuracy [20].

The MgH_2_ lattice was represented by a 3 × 3 × 1 supercell with 54 atoms that could accommodate different Ti, V, and Zr concentrations. In the Brillouin zone, 3 × 3 × 1 Monkhost–Pack grids were used to sample the reciprocal spaces. In contrast, the surface of Fermi structure and the electronic density of charges map were examined using a larger k-point mesh, measuring 9 × 9 × 9. The convergence process criteria for force and energy were 2 × 10^−5^ eV/atom, the highest Hellmann–Feynman ionic strength was restricted to 0.05 eV/Å, the highest stress level was capped at 0.1 GPa, and the maximum ion displacement was confined to 0.002 Å. All atoms were fully relaxed using a conjugate gradient algorithm. An atom was added or removed from the designed MgH_2_ supercell to create the defects, such as vacancies and interstitials. Structure relaxation was then carried out with specified lattice constants. Atomic locations inside the supercell were simultaneously given complete relaxation.

#### 2.1.2. Formation Energy and Atomic Structure

**Figure 3:**
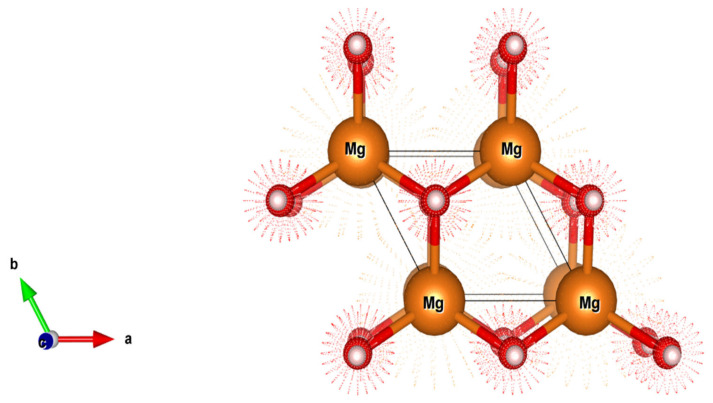
Magnesium hydride’s primitive cell (MgH_2_).

To determine the thermodynamic characteristics of our systems, using a relaxation approach, we first determined the equilibrium lattice parameters. In particular, by decreasing the total energy with respect to the volume of a 3 × 3 × 1 MgH_2_ supercell, we optimized the architectures (Figure 3). The energy associated with the formation of a charged defect (Xq) is contingent upon the atomic chemical potentials and the electronic chemical potential (Fermi level). It can be calculated using the following expression [21]:

(1) $$Mg+H_{2}\to \;MgH_{2}$$

(2) $$\left(1-x\right)Mg+xM+H_{2}\to \;{Mg}_{1-x}M_{x}H_{2}$$

The heat of formation for the reaction (Equation (2)) was calculated by subtracting the total energies of elemental Mg, the M atom (where M represents Ti, V, or Zr), and the H_2_ molecule from the total energy of the relaxed Mg_1−x_M_x_H_2_ hydride:

(3) $$∆H\left(MgH_{2}\right)=E_{tot}\left(MgH_{2}\right)-E_{tot}\left(Mg\right)-E_{tot}\left(H_{2}\right)$$

(4) $$∆H\left(M_{g0.9455}X_{0.0555}H_{2}\right)\;=E_{tot}\left(M_{g0.9455}X_{0.0555}H_{2}\right)-E_{tot}\left(Mg\right)-E_{tot}\left(X=Ti,\;V\;and\;Zr\right)-E_{tot}\left(H_{2}\right)$$

*E_tot_* represents the crystal supercell’s total energies with a defect X. Detailed results on thermodynamic properties are presented in Section 3.1.1.

### 2.2. Macroscopic Scale: Lumped Modeling for Hydrogen Storage Performance

**Figure 4:**
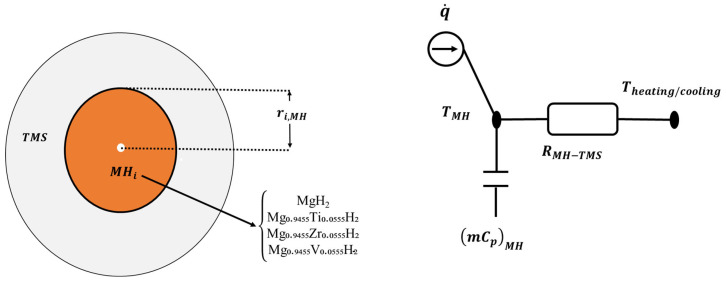
Scheme of the thermal network in the metal hydride reactor at the system level.

To simulate the dynamic behavior of the metal hydride storage with its thermal management system at the system level, lumped energy and mass balances are applied to each component of the reactor. These balances enable the determination of time-dependent variation in the reactor temperature and the progress of the hydrogenation process. More details about this approach can be found in our previous works [4,22]. In fact, this proposed lumped method considers the metal hydride reactor as a control volume with uniform temperature and a heat source. The latter is used to take into account the released heat during the exothermic reaction of the hydrogen absorption process. In addition to the reactor bed, the thermal management system, by using a heat transfer fluid, is also included in the system-level model. Figure 4 shows an analogous scheme for the mass and heat transport in the hydrogen storage reactor.

#### 2.2.1. Energy and Mass Balances

In order to predict the time-wise variation in the hydrogen storage temperature, the thermal management system temperature and the hydrogenation process, energy and mass balance at the system level are used and the following assumptions are considered:

- The hydrogen reactor has a uniform temperature and pressure.
- The hydrogen is considered as an ideal gas.
- The hydrogen reactor properties are independent of the temperature.
- The hydrogen storage tank is well insulted and exchanges heat only with the thermal management system.

Based on the previous assumptions, the governing equations can be given as the following:

- ***Energy balance:***

(5) $${\left(m_{H_{2}}C_{p_{H_{2}}}+m_{s}C_{p_{s}}\right)}_{1}\frac{dT_{MH}}{dt}=\frac{T_{TMS}-T_{MH}}{R_{MH-TMS}}+\dot{q}$$

In this expression, *T_MH_* and *T_TMS_* are the metal hydride temperature and used thermal management system temperature. $R_{MH-TMS}$ is thermal resistance between the thermal regulation system and the MH reactor. Its expression is given by:

(6) $$R_{MH-TMS}=\frac{1}{UA}$$

A is the heat transfer area between the heating/cooling system and the MH reactor, and U is the global heat transfer coefficient.

During the storage of the hydrogen in the reactor, the heat generated due to the exothermal reaction between the hydrogen (gas) and the metal (Mg) is given by as the following [4]:

(7) $$\dot{q}=\frac{{∆H}_{r}}{{MW}_{MH}}\frac{dm_{MH}}{dt}$$

${∆H}_{r}$ is the enthalpy reaction and its value depending on the used doped MgH_2_.

$\frac{dm_{MH}}{dt}$ is the metal hydride mass variation during the hydrogenation/dehydrogenation process and its expression is given in the following section.

- ***Mass balance***

The mass balance of the MH reactor, which expresses the evolution of the hydrogenation/dehydrogenation process, is given as a function of the kinetic reaction rate [23]:

(8) $$\frac{dm_{MH}}{dt}={r\cdot m}_{s}$$

- ***Reaction kinetic***

During the absorption/desorption of hydrogen in the metal, the process is affected by a number of physical parameters including hydrogen pressure, temperature, equilibrium pressure and activation energy. The general expression of the kinetic reaction can be expressed as a function of the previous physical parameters as [4,24,25]:

(9) $$r=C_{a}\;e^{\frac{-E_{a}}{RT_{MH}}}\;ln(\frac{P_{a}}{P_{eq}})\;(1-\;\frac{m_{MH}}{m_{s}})$$

where $P_{a}$ is the used inlet pressure at the MH reactor and $P_{eq}$ is the equilibrium pressure. The latter is given based on the Van’t Hoff law as follow [26,27]

(10) $$P_{eq}=P_{0}\;e^{\left(\frac{{∆H}_{r}}{RT_{MH}}-\frac{∆S}{R}\right)}$$

**Figure 5:**
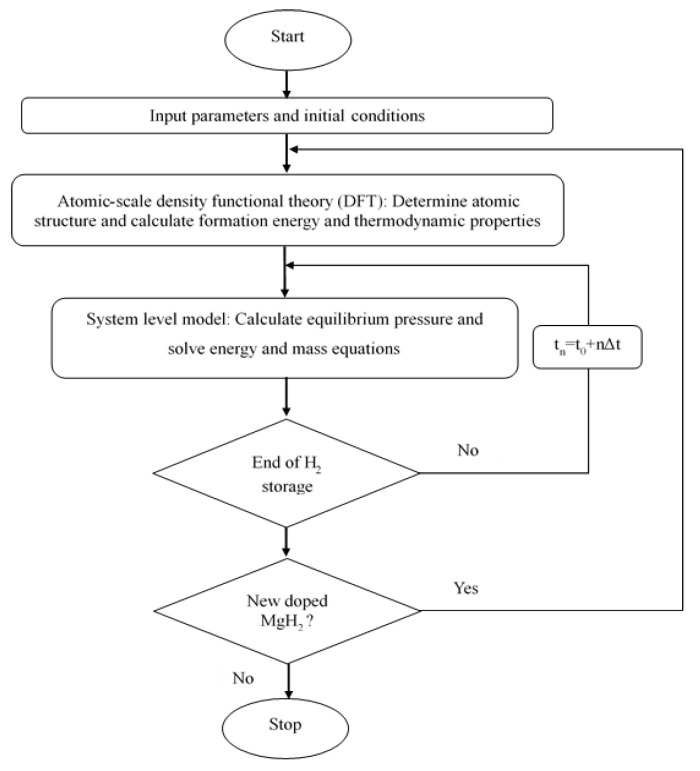
Flow chart of the coupling between atomic-scale and system-scale models.

It is interesting to note that the reaction enthalpy (${∆H}_{r}$) and the entropy (∆S) the studied doped MgH_2_ reactors are introduced in this lumped model based on output results from DFT calculations (See Section 2.1). A flow chart of the proposed coupling procedure between the micro-scale model and the system level model is given in Figure 5. In this multi-scale approach, for each doped MgH_2_ case study, DFT calculations are first carried out to determine the atomic structure, energetics, and thermodynamic properties of the material. These DFT-derived properties, are then integrated as input parameters into the lumped dynamic model to accurately simulate the hydrogen absorption behavior at the system level. This coupling ensures that material-scale modifications induced by doping are consistently reflected in the performance predictions of the reactor.

#### 2.2.2. Key Performance Indicators

Several key performance indicators (KPIs) are established in order to evaluate and compare the performance of MgH_2_-based hydrogen storage units under various doping techniques (Ti, V, Zr). These indicators provide quantifiable measurements for assessing the thermal and storage performance of hydrogen and are calculated using the results of the lumped dynamic simulations:

- *Hydrogenation Time (t_hyd_)*

Defined as the time required to reach a target hydrogen uptake fraction (e.g., 99% of the total capacity). It is strongly influenced by the reaction kinetics and thermal management efficiency. Its expression can be given as follow:

(11) $$t_{hyd}=\int_{X_{0}}^{X_{final}}\frac{dX}{r(P_{eq},T,P)}$$

where $r(P_{eq},T,P)$ is the kinetic reaction given as a function MH bed temperature, the equilibrium pressure and the used hydrogen pressure (See Equation (9)).

- *Maximum Reactor Temperature (T_max_)*

As mentioned previously, the MH temperature plays a critical role during the hydrogenation process. This KPI indicates the peak temperature reached during the hydrogen absorption process and it reflects the effectiveness of heat dissipation during the exothermic reaction.

- *Required heating/cooling thermal energy (Qth)*

This KPI measures the consumed thermal energy by the thermal management system during the hydrogenation process. Its expression is given by integrating the thermal power exchanged between the reactor and the thermal management system as follow:

(12) $$Q_{th}=\int_{0}^{t_{hyd}}{\dot{Q}}_{th}\left(t\right)dt$$

#### 2.2.3. Model Validation

**Figure 6:**
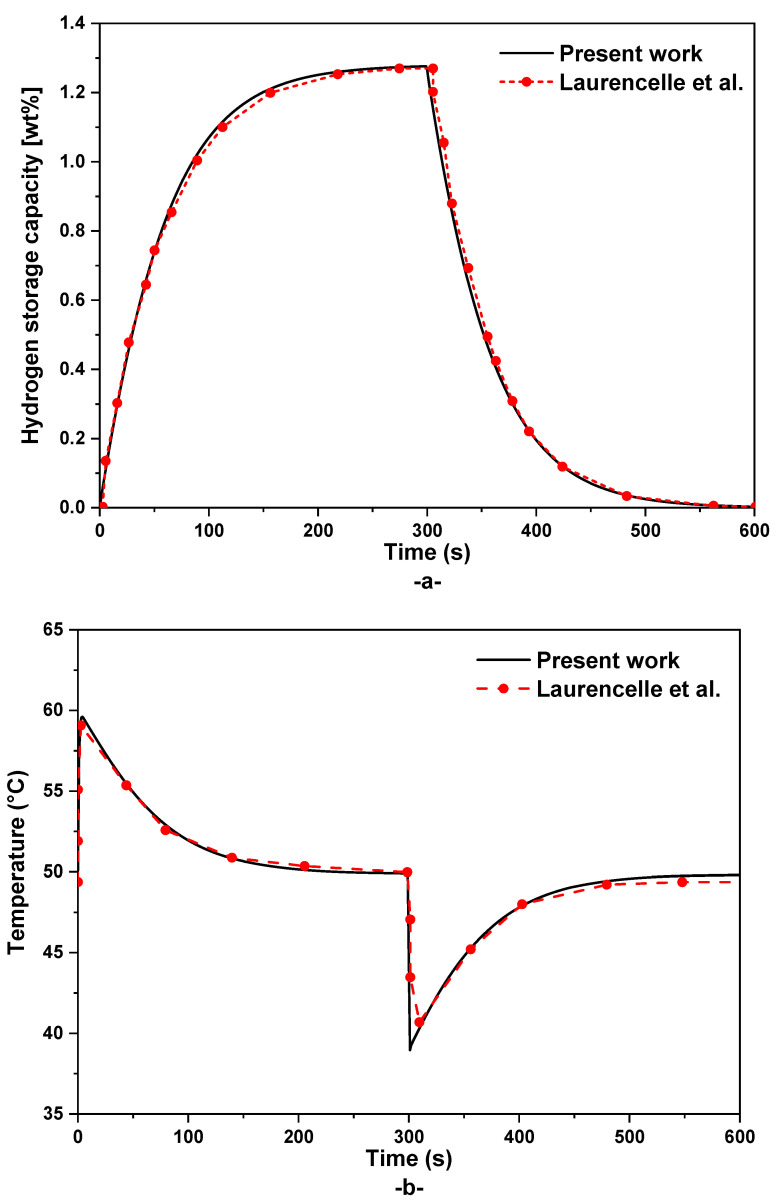
Comparison between measured and simulation results: (**a**) absorption/desorption of hydrogen and (**b**) average reactor temperature [28].

In this section, the proposed system level model is validated through comparing our numerical results with experimental results from the work of Laurencelle et al. [28]. This experimental study considers a MH reactor with a capacity of 25 g. This cylindrical reactor is heated/cooled using a water loop as TMS and the inlet hydrogen pressure is fixed at 12 bar. More details about this experimental work and the validation approach can be found in our previous work [4]. Based on obtained results in Figure 6, the proposed dynamic model is able to predict the real behavior of the MH hydrogenation and dehydrogenation process with a good accuracy (Figure 6a). In addition to the hydrogenation kinetic, the proposed system level-scale model is also able to predict the MH temperature during both hydrogenation/dehydrogenation processes (Figure 6b).

## 3. Results and Discussion

### 3.1. Microscopic Investigation: DFT Results

In this section, we employed the DFT to investigate the atomic-scale effect of Ti, V, and Zr doping on the electronic and thermodynamic properties of magnesium hydride (MgH_2_). The purpose of this study was to explore how dopant atoms disrupt the local atomic environment and change the fundamental properties of the material. The in-depth electronic and thermodynamic analyses that follow are based on the knowledge obtained through this atomic-level analysis.

#### 3.1.1. Electronic and Structural Parameters

Comprehending the electronic properties of materials is vital for understanding the mechanics of carrier transport, since it facilitates the distinction between metals, semiconductors, and insulators through the examination of their band gaps. Under ambient conditions, our MgH_2_ system has a rutile structure with a *P4*_2_*/mnm* space group. Experimentally, The primary lattice parameters of bulk MgH_2_ were found to be a = b = 4.501 Å and c = 3.010 Å. After optimization, the calculated equilibrium lattice parameters were a = b = 4.460 Å and c = 2.989 Å, closely matching experimental data and other computational results.

Figure 3 shows that magnesium is surrounded by six anionic hydrogen atoms. These hydrogen atoms can be separated into two groups:

- Two ‘apical’ hydrogen atoms are located above and below the magnesium, with an Mg-H distance of approximately 1.94 Å.
- Four ‘equatorial’ hydrogen atoms are located around the magnesium, forming a square, with a slightly larger Mg-H distance of about 2.03 Å.

The band gap width indicates the strength of the Mg-H bond: a smaller band gap suggests stronger electronic hybridization and less hydride stability, lowering the desorption temperature and reaction enthalpy. These modifications accelerate the kinetics of hydrogen sorption/desorption. Faster kinetics increase instantaneous thermal power during hydrogenation/dehydrogenation, whereas lower enthalpy requirements minimize overall thermal energy consumption.

This subsection examines the electronic structures and bonding properties of both pure MgH_2_ surfaces and those doped with Ti, V, and Zr. We analyze the total and partial densities of states (DOS) to understand the band energy, hybridization between different states, and charge transfer characteristics. This analysis helps determine how the addition of these transition metal elements affects hydrogen stability and desorption in MgH_2_.

**Figure 7:**
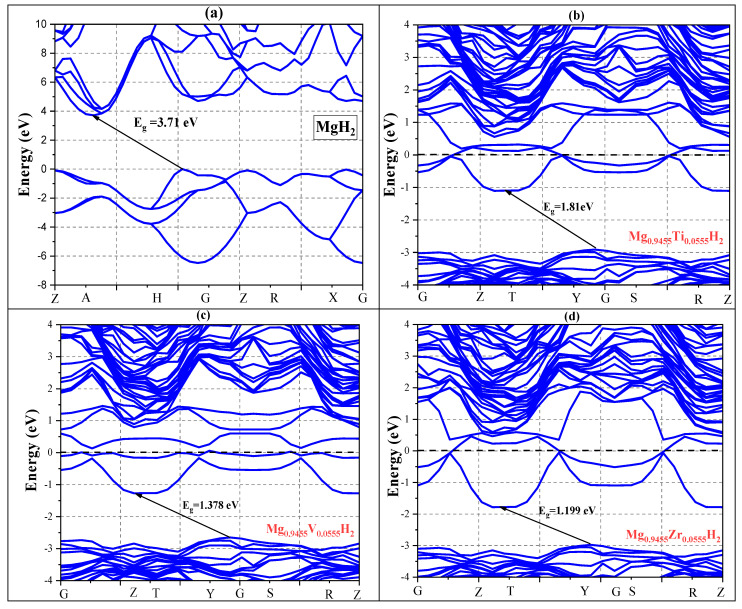
The band structures of (**a**) MgH_2_ pure and doped with (**b**) Ti, (**c**) V, and (**d**) Zr.

This analysis provides a detailed picture of the electronic state distribution and reveals the bonding and electronic structure of the material (Figure 7). Pure MgH_2_ reveals a non-metallic property, having a calculated band gap of 3.71 eV. While this is somewhat lower than the experimental value of 5.16 eV [29], it is consistent with the theoretical value of 3.6 eV referenced in [30]. The acknowledged limits of first-principles calculations utilizing PBE density functional theory are probably the cause of the discrepancy between our calculated and observed band gaps. As shown in Figure 7, doping with Ti, V, and Zr significantly reduces the band gap to 1.8 eV, 1.378 eV, and 1.199 eV, respectively, clearly demonstrating the impact of doping on the electronic structure.

#### 3.1.2. Charge Density Distribution

**Figure 8:**
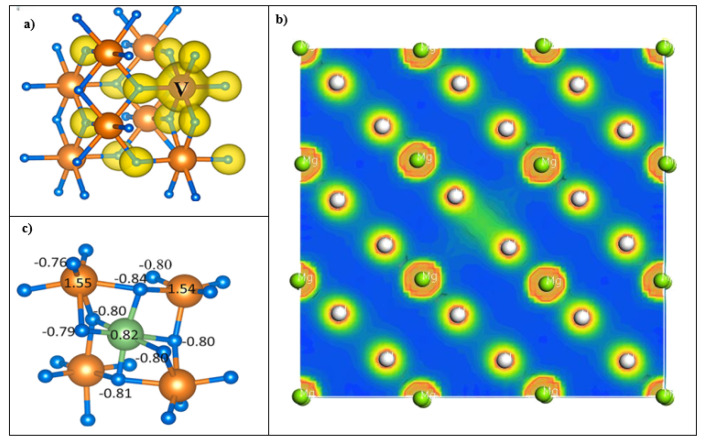
Charge density difference maps for (**a**), (**b**) Mg_0.9455_V_0.0555_H_2_ and (**c**) the calculated Bader charges (in unit of e). The orange, blue and green balls represent Mg, H and V, respectively.

An analysis of the electronic charge density for pure MgH_2_ and MgH_2_ doped with Ti, V, and Zr provides key insights into their bonding characteristics, particularly in cases of neutral charge distribution. The electron charge density was computed using the charge density difference approach from the CASTEP program, which is based on the GGA-PBE functional. This method was chosen because it emphasizes the redistribution of electronic density after Ti, V, and Zr doping, emphasizing the changes in Mg-H bonding properties and dopant-host interactions. Figure 8b shows a distinct localization of charge around the hydrogen atoms, indicating a strong covalent interaction with the Mg and V atoms. This is further evidenced by the directional charge localization observed between the H and V atoms. While the contribution of V atoms to the overall charge density is minimal, suggesting their ionic character, a mixed covalent-ionic bonding character exists between the hydrogen atoms and the dopants. This implies a weakening of the Mg-H bond compared to pure MgH_2_. These mixed interactions play a crucial role in stabilizing the structure by strengthening covalent bonds and weakening short-range interactions, ultimately influencing the material’s overall electronic and structural properties. Mg_0.9455_Ti_0.0555_H_2_ and Mg_0.9455_Ti_0.0555_H_2_ exhibit charge distribution patterns similar to Mg_0.9455_Ti_0.0555_H_2_, their patterns are not depicted.

**Figure 9:**
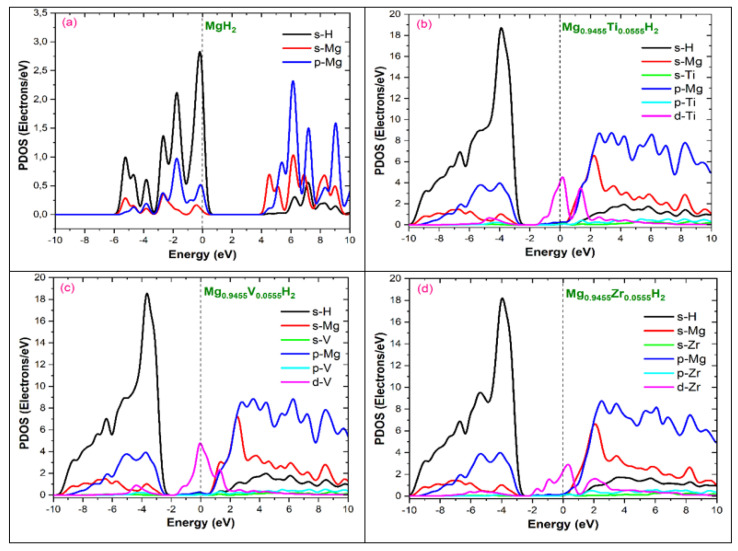
The Density of States (DOS) for (**a**) pure MgH_2_ and MgH_2_ doped with (**b**) Ti, (**c**) V, and (**d**) Zr. The vertical dashed line indicates the Fermi level E_F_.

Figure 9a clearly shows hybridization peaks in the valence and conduction bands of pure MgH_2_, indicating strong Mg-H bond orbital interactions. The conduction band is primarily characterized by Mg-s and p states. These strong interactions likely contribute to the high desorption temperature of MgH_2_. In contrast, the DOS plots for Ti, V, and Zr-doped MgH_2_ (Figure 9b–d, respectively) reveal defect states within the band gap. These defect states arise from interactions between H and the Ti, V, or Zr atoms. Were the defect states’ refer to localized electronic energy levels introduced into the band gap of MgH_2_ as a result of dopant atoms (Ti, V, or Zr) and the associated lattice distortions or vacancies. These states arise from the perturbation of the host crystal’s electronic structure and can influence charge transfer and, consequently, the kinetics of hydrogen absorption/desorption.

Figure 9b–d shows weak hybridization between the (V, Ti, and Zr) d orbitals and the neighboring H s orbital, as evidenced by the alignment of their respective peaks. In addition, the Mg-H hybridization within the high-energy segment of the valence band is lessened. The V d and H s orbitals show the highest degree of hybridization, reflecting the most robust chemical interaction between V and H. While pure MgH_2_ displays strong Mg-H hybridization, this hybridization weakens significantly in the doped structures, with a near absence of MgH_2_ hybrid peaks. These strong inter-state hybridizations confirm the high stability of the doped materials, consistent with the calculated ΔH values. Our findings are consistent with [8].

#### 3.1.3. Thermodynamic Properties

(a) Hydrogen desorption energy

To demonstrate the changes made to the thermodynamic properties, by doping MgH_2_ with the transition elements Ti, V and Zr. We examined the hydrogen desorption activation energy (*E_d_*) for the 3 × 3 × 1 supercell using the equation below [7]:

*E_d_* = *E*(*MgH*_2_ + x) + x/2 *E*(*H*_2_) − *E*(*MgH*_2_) (13)

*E(MgH*_2_*+ xVH)* represents the energy of the MgH_2_ in which an H_2_ atom is taken away from the relaxed MgH_2_, *E(H*_2_*)* refers to the energy associated with the H_2_ molecule at 0 K, and *E(MgH*_2_*)* indicates the energy of the MgH_2_ surface before the process of dehydrogenation. Our calculated value of 109.21 kJ/mol agrees with previous theoretical calculations [31], although it is below the experimental values of approximately 143.0 and 160.6 kJ/mol [32]. This high desorption energy is attributed to the strong ionic bonds between the hydrogen atoms.

Table 1 shows the evaluated hydrogen desorption energies for pure and Ti, V, and Zr-doped MgH_2_ at various doping levels. The desorption energies for the doped systems range from 69 ➔ 78 kJ/mol, significantly lower than that of pure MgH_2_. MgVH_2_ exhibits the lowest desorption energy, likely due to the reduced number of H-Mg ionic bonds, as each hydrogen atom is coordinated by only two Mg atoms.

(b) Formation enthalpy

The enthalpy of formation (ΔH) influences the heat of hydrogenation and dehydrogenation reactions. It is computed by subtracting the total energy of the reactants and products of the reaction.

Δ*H* = ∑*E*_tot_(products) − ∑*E*_tot_(reactants) (14)

Equations (3), (4), and (14) have been used to determine the formation enthalpies for the systems. Our calculated enthalpy of formation for pure MgH_2_ is −63.961 kJ/mol H_2_, which closely agrees with previous theoretical and experimental values: −63.68 kJ/mol H_2_ [33], ~−75 kJ/mol H_2_ [34], ~−62.01 kJ/mol H_2_ [8], 88.571 kJ/mol H_2_ [35], and −82 kJ/mol H_2_ [36]. The calculated enthalpies of formation for Mg_0.9455_V_0.0555_H_2_, Mg_0.9455_Zr_0.0555_H_2_, and Mg_0.9455_Ti_0.0555_H_2_ are −50.117, −51.192, and −51.212 kJ/mol H_2_, respectively. The enthalpies of formation for the doped structures are lower than those of pure MgH_2_, suggesting a reduction in stability and a decrease in the dehydrogenation temperature. Doping MgH_2_ with Ti, V, and Zr alters its thermodynamic properties, including the enthalpy of formation and the desorption temperature, which arise from the structural and electronic changes induced within the lattice. Due to their smaller atomic radii and distinct electronic properties, each dopant induces crystalline distortions that disrupt electron distribution and weaken Mg-H bonds by modifying the local electron density, thereby promoting hydrogen release. The Mg_0.9455_V_0.0555_H_2_ system exhibits the highest average neutral hydrogen removal energy among the three doped materials, consistent with the Density of States (DOS) analysis presented in Section 3.1.1.

(c) Desorption temperature of the pure and doped MgH_2_

The temperature of the desorption process is a crucial factor in assessing the practical applicability of materials for hydrogen storage, directly impacting the conditions required for hydrogen release from MgH_2_. This temperature can be evaluated using the Van’t Hoff equation [37]:

(15) $$\frac{P_{H_{2}}}{P_{0}}=exp\left(-\frac{\Delta H}{RT}+\frac{\Delta S}{R}\right)$$

The pressure at equilibrium for H_2_ is denoted as *P_H_*_2_, with the ambient pressure labeled as *P*_0_, the gas constant as R, the absolute temperature as *T*, and the changes in enthalpy and entropy that occur during the hydrogen desorption reaction represented by Δ*H* and Δ*S*, respectively. The desorption energy of molecular hydrogen in our calculation’s desorption reaction is represented by Δ*H*, and the hydrogen pressure is comparatively small. Since the typical pressure in the atmosphere (*P*_H2_ = *P*_0_ = 1 atm) is more closely associated with actual operating circumstances, we are particularly interested in it. In these circumstances, the desorption temperature can be easily calculated using the formula: *Td* = Δ*H/*Δ*S*.; The change in entropy, Δ*S*, is estimated to be 130 J mol^−1^ K^−1^ for gaseous H_2_ in the context of metal hydrides [34].

The estimated desorption temperature for undoped MgH_2_ is 487.37 K, consistent with previous theoretical studies [8,37]. The estimated desorption temperatures for the doped systems at 1 atm are 391 K for both Ti- and Zr-doped MgH_2_, and 383 K for V-doped MgH_2_. These lower temperatures indicate that adding Ti, V, and Zr significantly reduces the energy barrier for dehydrogenation.

**Figure 10:**
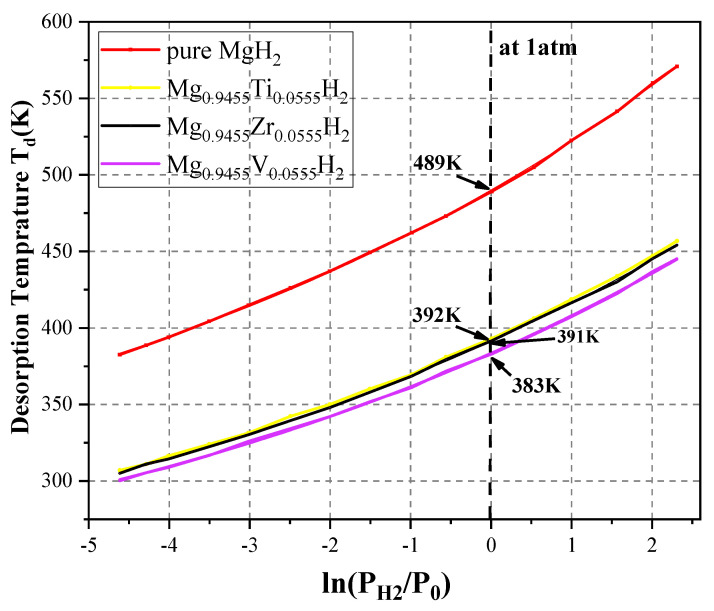
Variation in hydrogen desorption temperature relative to equilibrium pressure.

Figure 10 illustrates the change in hydrogen desorption temperature (Td) as a function of equilibrium pressure (ranging from 0.01 ➔ 10 atm) for both undoped MgH_2_ and MgH_2_ doped with Ti, V, and Zr. As expected, T_d_ increases with increasing pressure. Notably, the curves for the three doped compounds lie below the curve for pure MgH_2_, confirming that doping with Ti, V, and Zr lowers the desorption temperature. The relative effectiveness of these dopants in reducing Td follows this order: Mg_0.9455_Ti_0.0555_H_2_ > Mg_0.9455_Zr_0.0555_H_2_ > Mg_0.9455_V_0.0555_H_2_. These trends are consistent with previous findings reported in Ref. [8].

### 3.2. Macroscopic Investigation: System-Scale Modeling Results

**Figure 11:**
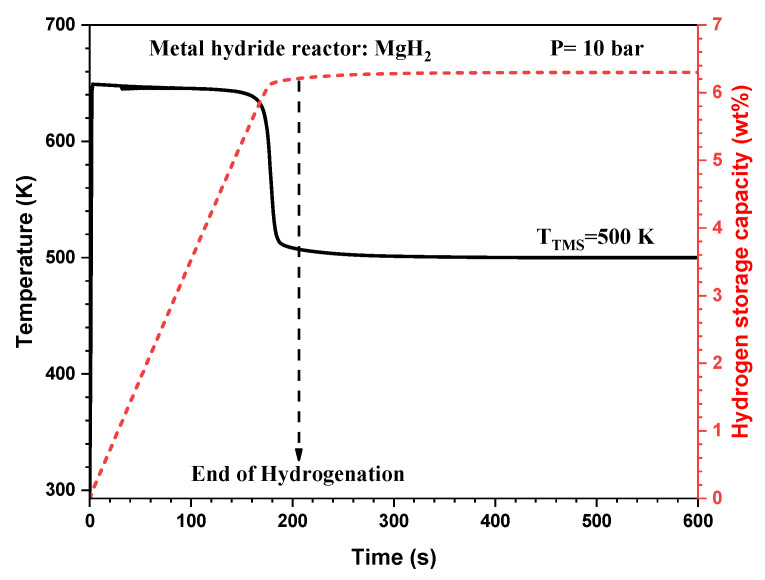
Time-wise variation in the hydrogenation process and the average reactor temperature using pure MgH_2_.

At the system level, the proposed lumped model is developed based on energy and mass balances to simulate the real dynamic behavior of the MgH_2_-based hydrogen storage reactor under various doping conditions. Furthermore, this model is completed by the hydrogen kinetic reaction expression to predict the hydrogenation/dehydrogenation process. Figure 11 shows the evolution of the hydrogenation capacity and the reactor temperature during the hydrogenation process of the MgH_2_-based hydrogen storage reactor. It should be noted that a thermal management system is integrated to regulate the reactor temperature with a control setpoint fixed at 500 K.

It can be shown clearly that at the onset of the hydrogenation process, the reactor temperature rises rapidly until reaching a temperature of 649 K where it becomes nearly stable. This temperature increase is attributed to the exothermic nature of the hydrogen absorption reaction, which generates significant thermal energy. The used heat transfer fluid effectively absorbs and dissipates this heat, maintaining the reactor temperature within the desired operating range. This result also illustrates the evolution of the hydrogenation process and indicates the time needed to fully charge the hydrogen rector. In this MgH_2_-based reactor, the hydrogenation time is about 3.5 min under the specified operating condition.

**Figure 12:**
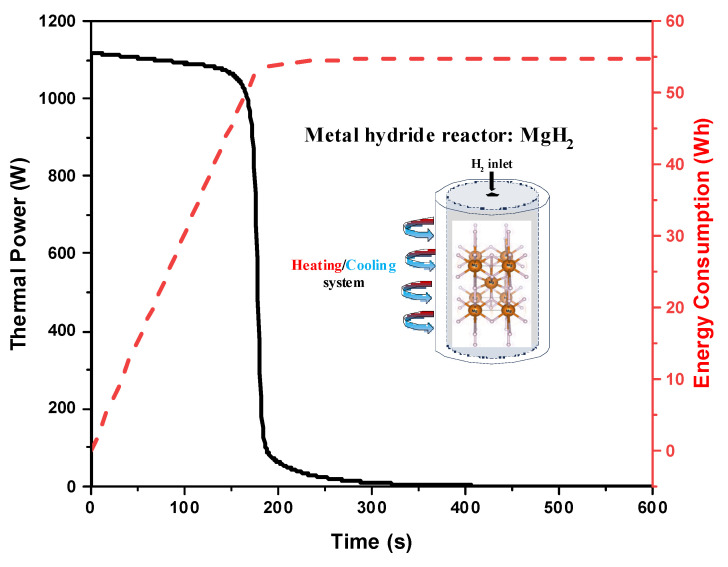
Instantaneous thermal power and consumed thermal energy by the thermal management system.

Through analyzing the instantaneous removed thermal power from the MgH_2_-based reactor (Figure 12), it can be observed that high amount of thermal power is removed during the beginning of the hydrogenation process and it about 1.12 kW. This high thermal load corresponds to the initial stage of hydrogen absorption, where the exothermic reaction rapidly generates heat. This removed thermal power decreases with the progress of the hydrogenation process until the end of the process where the reactor bed temperature becomes equal to the heat transfer fluid temperature. This dynamic behavior influences the thermal heating/cooling energy consumption as shown in the same figure. The cumulated removed thermal power from the MgH_2_-based reactor increases linearly with the hydrogenation process and the final thermal energy consumption of this system is about 55 Wh to store 1.575 g of hydrogen under the specific operation conditions. It is important to note that this energy value accounts only for the heat removed from the reactor and does not include the energy required to heat up or maintain the heat transfer fluid at the setpoint temperature of 500 K. Based on this, the specific thermal energy removal is about 34.9 Wh/g H_2_.

**Figure 13:**
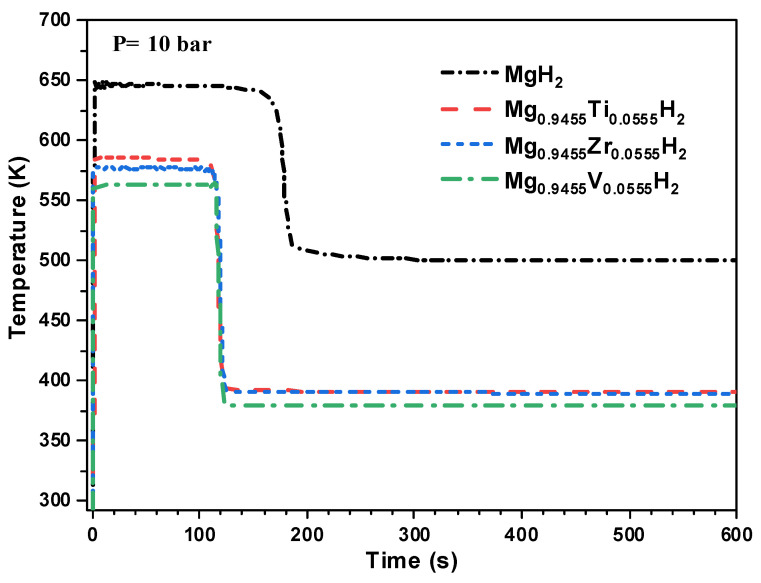
Impact of Ti, V, and Zr doping on the temperature evolution of the MgH_2_-based hydrogen storage reactor during the hydrogenation process at 10 bar.

Figure 13 presents the time-wise variation in the reactor temperature during the hydrogenation process for both pure and doped MgH_2_ systems using Ti, V, and Zr as doping elements. The reference case, corresponding to pure MgH_2_, exhibits a sharp temperature increase due to the highly exothermic nature of the absorption reaction, reaching a peak temperature of approximately 653 K. In contrast, all doped systems show significantly lower maximum temperatures: around 585 K for Ti-doped, 575 K for Zr-doped, and 563 K for V-doped MgH_2_. This trend reflects an improved thermal behavior, which is attributed to reduced reaction enthalpy and enhanced hydrogen sorption kinetics due to doping.

These macroscopic observations are in good agreement with micro-scale (DFT simulations) which showed that doping MgH_2_, particularly with vanadium, leads to a substantial decrease in both the reaction enthalpy and the activation energy required for hydrogen absorption (see Table 1). This reduction is translated in the macro-scale dynamic model by the reduction in the maximal reached temperature in the hydrogen storage reactor, during the hydrogenation process. Moreover, the use of doped MgH_2_ materials results in a lower required operating temperature of the heat transfer fluid (HTF) to manage the thermal load. While the pure MgH_2_ system requires the HTF to be maintained at 500 K, the necessary HTF temperatures are reduced to 391 K, 390 K, and 379 K for the Ti-, Zr-, and V-doped systems, respectively.

**Figure 14:**
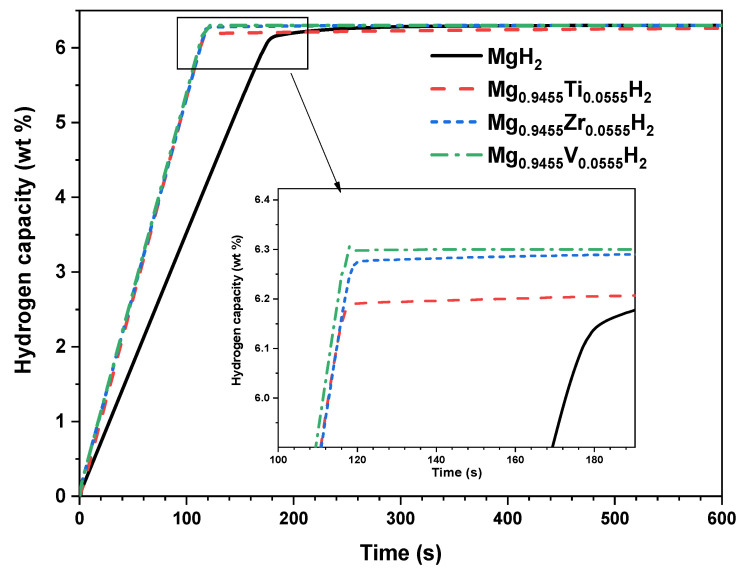
Impact of Ti, V, and Zr doping on the hydrogen storage capacity evolution.

To show the impact of the doping element on the hydrogenation process, the hydrogen capacity evolution of both pure and doped MgH_2_ is given in Figure 14. This result clearly shows that doping the MgH_2_ reactor is advantageous and leads to accelerating the hydrogenation kinetics, thereby reducing the total charging time of the reactor.

Compared to the reference case of pure MgH_2_, the hydrogenation time is reduced by approximately 21% with Ti doping, 40% with Zr doping, and 42% with V doping. This improvement in reaction kinetics is consistent with the lower activation energies and enhanced thermodynamic behavior of the doped materials, as previously predicted by DFT simulations (see Table 1). The accelerated hydrogen uptake observed at the system scale confirms the beneficial role of doping elements in improving both the reaction rate and operational performance of MgH_2_-based hydrogen storage systems.

**Figure 15:**
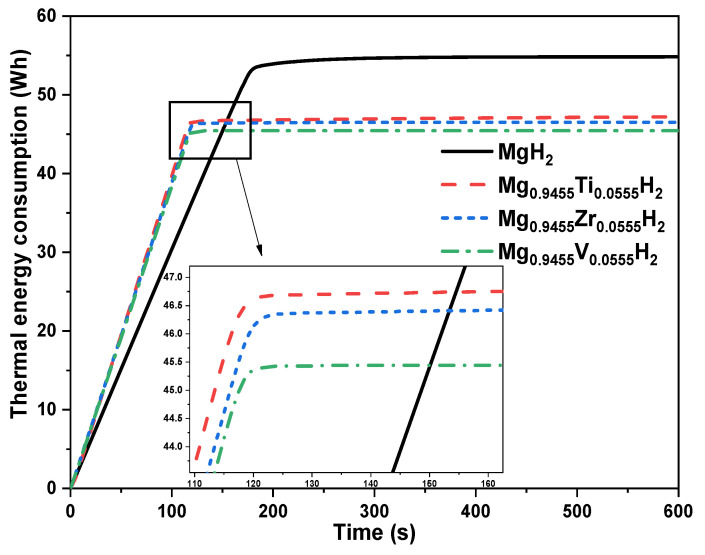
Influence of Ti, V, and Zr doping on consumed thermal energy by the thermal management system.

Figure 15 illustrates the influence of doping on the consumed thermal energy during the hydrogenation process. It is evident that using pure Mg as the metal hydride for hydrogen storage leads to increase significantly the thermal energy consumption by the thermal regulation system. In contrast, the incorporation of doping elements such as Ti, Zr, and V significantly reduces the energy needed to regulate the reactor temperature throughout the absorption phase.

Among the evaluated cases, Vanadium-doped MgH_2_ exhibits the lowest energy demand. Specifically, the thermal energy required to complete the hydrogenation process is approximately 55 Wh for the pure MgH_2_ reactor, compared to 47 Wh, 46.5 Wh, and 45.5 Wh for the Ti-, Zr-, and V-doped systems, respectively. This energy is primarily used to maintain optimal thermal conditions during the exothermic absorption process and to ensure full hydrogen storage, with a maximum capacity of 1.575 g of H_2_ in each case. As a result, the specific thermal energy removal, which is defined as the energy removed per gram of stored hydrogen, decreases from 34.9 Wh/g H_2_ for pure MgH_2_ to 29.8 Wh/g H_2_ with Ti doping, 29.5 Wh/g H_2_ with Zr doping, and 28.8 Wh/g H_2_ with V doping. These reductions highlight the benefit of doping in improving the overall thermal efficiency of metal hydride-based hydrogen storage systems.

These obtained key performance indicators, including hydrogenation time, percentage reduction, thermal energy consumption, and specific thermal energy removal, are summarized in Table 2. This comparison highlights the beneficial effect of Ti, Zr, and V doping on improving both the thermal and kinetic performance of MgH_2_-based hydrogen storage reactors.

It is important to acknowledge that while the present study highlights improvements in hydrogenation kinetics and thermal management through transition metal doping, other critical aspects such as cycling stability, phase stability, and long-term reversibility were not explicitly investigated in this work. These factors are indeed crucial for the commercial adoption of MgH_2_-based storage materials, as repeated hydrogenation/dehydrogenation cycles may lead to particle coarsening, structural degradation, or loss of capacity. Experimental studies in the literature have consistently identified cycling degradation and phase transformations as key barriers to practical implementation. The current modeling framework can be extended in future work to incorporate degradation phenomena, for example, by introducing cycle-dependent kinetic or thermodynamic parameters, thereby enabling a more comprehensive assessment of material and system performance under realistic operating conditions.

## 4. Conclusions

The multi-scale investigation indicates that magnesium hydride’s (MgH_2_) ability to store hydrogen is greatly improved by doping it with transition metals including Ti, Zr, and V. By lowering the energy barriers related to hydrogen absorption and desorption, these dopants enhance the thermodynamic properties of MgH_2_ at the atomic scale, as confirmed by DFT analysis. The doped materials show enhanced hydrogenation kinetics and improved thermal management at the system level. With the lowest specific thermal energy removal of 28.8 Wh/g H_2_ and a 17% reduction in energy consumption, V-doped MgH_2_ outperforms the others in terms of thermal efficiency. The importance of the suggested multi-scale modeling technique for the design and optimization of enhanced hydrogen storage systems is demonstrated by these results, which show a strong link between atomic-scale alterations and macroscopic performance improvements.

The findings highlight the critical role of doping in the development of tailored materials and the system-level optimization of hydrogen storage technologies. Future research could focus on exploring a wider range of dopant elements, their concentrations, and the potential synergistic effects of co-doping. In fact, the proposed multi-scale framework can be readily extended to investigate co-doping and multi-component nanocomposites, which are expected to provide synergistic improvements in hydrogen storage performance and will be the subject of future studies. To translate these results into practical and commercially viable storage systems, it will also be essential to combine experimental validation with long-term cycling performance and to investigate the scalability of the approach under diverse operating conditions.

## Figures and Tables

**Table 1:** Summary of formation enthalpy, the desorption temperature and the activation energy calculated as a function of Equations (2), (3), and (13) for pure and doped MgH_2_ and for comparative studies.

Structures	ΔH (kJ/(mol H_2_))	T_d_ (K)	Activation Energy (kJ/mol)
MgH_2_	−63.961	489.37	109.21
Mg_0.9455_Ti_0.0555_H_2_	−51.212	391.82	78.24
Mg_0.9455_V_0.0555_H_2_	−50.117	383.45	69.11
Mg_0.9455_Zr_0.0555_H_2_	−51.192	391.67	75.49

**Table 2:** Summary of system-level hydrogenation performance indicators for pure and doped MgH_2_ Reactors.

Material	Hydrogenation Time (min)	Reduction in Time (%)	Thermal Energy Consumption (Wh)	Energy Reduction (%)	Specific Thermal Energy Removal (Wh/g H_2_)
Pure MgH_2_	3.5	—	55.0	—	34.9
Ti-doped MgH_2_	2.76	21%	47.0	14.5%	29.8
Zr-doped MgH_2_	2.10	40%	46.5	15.5%	29.5
V-doped MgH_2_	2.03	42%	45.5	17.3%	28.8

## Data Availability

Data is contained within the article.

## References

[1] Alobaid A., Kamil M., Khalil K.A. (2025). Metal hydrides for solid hydrogen storage: Experimental insights, suitability evaluation, and innovative technical considerations for stationary and mobile applications. Int. J. Hydrogen Energy.

[2] Lebrouhi B.E., Djoupo J.J., Lamrani B., Benabdelaziz K., Kousksou T. (2022). Global hydrogen development—A technological and geopolitical overview. Int. J. Hydrogen Energy.

[3] Hong H., Guo H., Cui Z., Ball A., Nie B. (2024). Structure modification of magnesium hydride for solid hydrogen storage. Int. J. Hydrogen Energy.

[4] Lamrani B., Alqahtani R.T., Ajbar A., Kousksou T. (2025). Resistance-capacitance dynamic model for hydrogen storage in metal hydrides with phase change material as thermal management system. Energy Convers. Manag. X.

[5] Zavaliy I., Berezovets V., Denys R., Kononiuk O., Yartys V. (2023). Hydrogen absorption-desorption properties and hydrolysis performance of MgH_2_-Zr_3_V3O_0.6_H_x_ and MgH_2_-Zr_3_V_3_O_0.6_H_x_-C composites. J. Energy Storage.

[6] Xiao H., Qian F., Zhang X., Hu H., Tang R., Hu C., Zhou W., He X., Pu Z., Ma C. (2024). Effect of Ce_0.6_Zr_0.4_O_2_ nanocrystals on boosting hydrogen storage performance of MgH_2_. Chem. Eng. J..

[7] Li Z., Lu Y., Wang J., Chen Y., Li Q., Pan F. (2024). Improved hydrogen storage kinetics of MgH_2_ using TiFe_0.92_Mn_0.04_Co_0.04_ with in-situ generated α-Fe as catalyst. Mater. Rep. Energy.

[8] Han B., Wang J., Tan J., Ouyang Y., Du Y., Sun L. (2024). First-principles study on the dehydrogenation thermodynamics and kinetics of Ti, Zr, V and Nb doped MgH_2_. J. Energy Storage.

[9] Lobo R., Alvarez N., Shanov V. (2021). Hydrogen nanometrology in advanced carbon nanomaterial electrodes. Nanomaterials.

[10] Firlej L., Kuchta B., Walczak K., Journet C. (2021). Hydrogen storage in pure and boron-substituted nanoporous carbons—Numerical and experimental perspective. Nanomaterials.

[11] Elman R.R., Kurdyumov N., Laptev R.S., Kudiiarov V.N. (2025). The influence of single-walled carbon nanotubes additives on the structure and hydrogenation behavior of magnesium hydride. J. Energy Storage.

[12] Gaztañaga F., Luna C.R., Sandoval M., Macchi C., Jasen P. (2016). Geometric, Electronic, and Magnetic Properties of MgH_2_: Influence of Charged Defects. J. Phys. Chem. C.

[13] Germán E., Luna C., Marchetti J., Jasen P., Macchi C., Juan A. (2014). A DFT study of dopant (Zr, Nb) and vacancies on the dehydrogenation on MgH_2_ (001) surface. Int. J. Hydrogen Energy.

[14] Luna C.R., Germán E., Macchi C., Juan A., Somoza A. (2013). On the perfect MgH_2_(–Nb,–Zr) systems and the influence of vacancy-like defects on their structural properties. A self-consistent first principle calculations study of the electron and positron parameters. J. Alloys Compd..

[15] Reddad K., Labrim H., Zejli D., El Bouayadi R. (2024). Enhancing hydrogen desorption in MgH_2_: A DFT study on the effects of copper and zinc doping. Int. J. Hydrogen Energy.

[16] Clark S.J., Segall M.D., Ii C.J.P., Hasnip P.J., Probert M.I.J., Refson K., Payne M.C. (2005). First principles methods using CASTEP. Z. Kristallogr./Cryst. Mater..

[17] Segall M.D., Lindan P.J.D., Probert M.J., Pickard C.J., Hasnip P.J., Clark S.J., Payne M.C. (2002). First-principles simulation: Ideas, illustrations and the CASTEP code. J. Phys. Condens. Matter.

[18] Perdew J.P. (1991). Generalized gradient approximations for exchange and correlation: A look backward and forward. Phys. B Condens. Matter.

[19] Bende D., Wagner F.R., Sichevych O., Grin Y. (2017). Chemical Bonding Analysis as a Guide for the Preparation of New Compounds: The Case of VIrGe and HfPtGe. Angew. Chem..

[20] Perdew J.P., Ruzsinszky A., Csonka G.I., Vydrov O.A., Scuseria G.E., Constantin L.A., Zhou X., Burke K. (2008). Restoring the density-gradient expansion for exchange in solids and surfaces. Phys. Rev. Lett..

[21] Monkhorst H.J., Pack J.D. (1976). Special points for Brillonin-zone integrations. Phys. Rev. B.

[22] Lebrouhi B.E., Lamrani B., Ouassaid M., Abd-Lefdil M., Maaroufi M., Kousksou T. (2022). Low-cost numerical lumped modelling of lithium-ion battery pack with phase change material and liquid cooling thermal management system. J. Energy Storage.

[23] Talagañis B.A., Meyer G.O., Aguirre P.A. (2011). Modeling and simulation of absorption-desorption cyclic processes for hydrogen storage-compression using metal hydrides. Int. J. Hydrogen Energy.

[24] Bhouri M., Bürger I. (2017). Numerical investigation of H_2_ absorption in an adiabatic high-temperature metal hydride reactor based on thermochemical heat storage: MgH_2_ and Mg(OH)_2_ as reference materials. Int. J. Hydrogen Energy.

[25] Jain I.P., Lal C., Jain A. (2010). Hydrogen storage in Mg: A most promising material. Int. J. Hydrogen Energy.

[26] Klopčič N., Grimmer I., Winkler F., Sartory M., Trattner A. (2023). A review on metal hydride materials for hydrogen storage. J. Energy Storage.

[27] Hassan I.A., Mohammed R.H., Ramadan H.S., Saleh M.A., Cuevas F., Hissel D. (2023). Performance evaluation of a novel concentric metal hydride reactor assisted with phase change material. Appl. Therm. Eng..

[28] Laurencelle F., Goyette J. (2007). Simulation of heat transfer in a metal hydride reactor with aluminium foam. Int. J. Hydrogen Energy.

[29] Van De Walle C.G., Neugebauer J. (2004). First-principles calculations for defects and impurities: Applications to III-nitrides. J. Appl. Phys..

[30] Sprunger P.T., Plummer E.W. (1991). An experimental study of the interaction of hydrogen with the Mg(000 1) surface. Chem. Phys. Lett..

[31] Bouhadda Y., Rabehi A., Bezzari S.T.-C. (2007). First-principle calculation of MgH_2_ and LiH for hydrogen storage. J. Renew. Energ..

[32] Wu G., Zhang J., Li Q., Wu Y., Chou K., Bao X. (2010). Dehydrogenation kinetics of magnesium hydride investigated by DFT and experiment. Comput. Mater. Sci..

[33] Fernández J.F., Sánchez C.R. (2003). Simultaneous TDS-DSC measurements in magnesium hydride. J. Alloys Compd..

[34] Park M.S., Janotti A., Van de Walle C.G. (2009). Formation and migration of charged native point defects in MgH_2_: First-principles calculations. Phys. Rev. B.

[35] Bogdanovic B., Bohmhammel K., Christ B., Reiser A., Schlichte K., Vehlen R., Wolf U. (1999). Thermodynamic investigation of the magnesium-hydrogen system. J. Alloys Compd..

[36] Bahou S., Labrim H., Ez-Zahraouy H. (2023). Role of vacancies and transition metals on the thermodynamic properties of MgH_2_: Ab-initio study. Int. J. Hydrogen Energy.

[37] Pozzo M., Alfè D. (2008). Structural properties and enthalpy of formation of magnesium hydride from quantum Monte Carlo calculations. Phys. Rev. B Condens. Matter. Mater. Phys..

